# Refined Auditory Brainstem Response Measurement Identified Potential Models of Congenital Deafness in Laboratory Mouse Strains

**DOI:** 10.31662/jmaj.2018-0067

**Published:** 2019-07-08

**Authors:** Akiko Furutani, Youji Asama, Hiroyuki Sasaki, Shigenobu Shibata

**Affiliations:** 1 Asama Institute, Ibaraki, Japan; 2 Laboratory of Physiology and Pharmacology, School of Advanced Science and Engineering, Waseda University, Tokyo, Japan

**Keywords:** hearing loss, hearing tests, Evoked Potentials, Auditory, Brain Stem, Mice

## Abstract

**Introduction:**

To detect congenital hearing loss in mice, an objective measure is needed other than mouse behavioral observation. This study aimed to refine the methodology of auditory brainstem response measurement and identify potential congenital hearing loss models in laboratory mouse strains.

**Methods:**

Mice were anesthetized and fitted with head electrodes. Each mouse underwent four ABR measurements according to four testing conditions: A - no chamber; conventional tone; B - chamber; conventional tone; C - no chamber; short tone; D - chamber; short tone. Potential congenital hearing loss models were identified using 10 mice from each strain (C57BL, BALB/c, CH3, ICR, and ddY) through sound-attenuated ABR measurements with short-tone bursts. Potential congenital hearing loss models exhibited hearing thresholds ≥30 dB in both ears. Data were analyzed for normal distribution and variance homogeneity using the D’Agostino-Pearson/Kolmogorov-Smirnov and F value tests, respectively. One-way analysis of variance (ANOVA), with the Tukey-Kramer test, was used to conduct parametric analysis, and the Kruskal-Wallis/Friedman test, with a Dunn’s test for post hoc analysis, was used to perform non-parametric analysis.

**Results:**

The simultaneous use of a sound-attenuating chamber and short-tone bursts provided clearly defined wave patterns, even at lower sound intensities. Inbred strains, especially C57BL/6 sub-strains, constitute suitable congenital hearing loss models.

**Conclusions:**

Our study shows that environmental factors should be addressed in animal studies of hearing function. Potential congenital hearing loss models may be found amongst commercially available inbred strains.

## Introduction

Hearing loss is the most common sensory disorder, affecting approximately 70 million people worldwide ^(1)^. Three of every 1,000 newborns experience hearing loss; one of these three exhibits congenital deafness ^(2), (3)^. Common consequences of congenital deafness include speech and communication disorders, delay in language understanding, educational disadvantage, and social segregation and stigmatization ^(4)^; thus, early detection is essential for improving the quality of life for affected children.

Environmental causes, such as trauma, toxicity, or premature birth, contribute to 40%-50% of hearing loss cases; 50%-60% are caused by genetic factors ^(1)^. Genetic hearing loss can be classified as syndromic or non-syndromic, depending on whether it is related to other clinical issues. Many pediatric cases are non-syndromic ^(5)^; these can be categorized as autosomal recessive (75%-80%), autosomal dominant (15%-25%), or X-linked or mitochondrial (both comprising 1%-2% of all cases) ^(6)^.

Mouse models are vital to understanding of hearing loss. Large numbers of mice can be screened easily for hearing phenotyping of mouse mutants; such screening led to the identification of several deafness genes, each of which contributed to understanding human hearing impairments ^(7)^. Mice and humans share remarkable similarities with respect to the auditory system; using mouse models allows histopathological and ultrastructural studies, which are not feasible in human patients ^(8)^. Mutant mouse models of hereditary hearing loss have helped to identify genes involved in the inner ear’s development or function; this has allowed better understanding of the genetic basis of hearing ^(9)^, as well as the genetic and physiological mechanisms underlying early- and late-onset human deafness ^(10)^.

A standard test to evaluate hearing loss in humans and animals is the automated measurement of auditory brainstem responses (ABRs)―electrical potential signals emanating from the brain scalp upon presentation of a sound stimulus ^(4)^; during presentation of sound stimulus, the brain elicits electric signals, known as auditory evoked potentials (AEPs), of which ABRs comprise the earlier portion (0-12 msec) ^(4)^. ABRs comprise several waves and peaks, typically identified by Roman numerals, of which waves I, III, and IV are the most clinically significant ^(11)^. ABRs can be measured non-invasively, via electroencephalography (EEG), and used to assess a subject’s hearing threshold. They are interpreted by measurements of peak and inter-peak latencies, calculated from pre-recorded wave patterns; visual scoring is the most common interpretation method and can be subjective ^(12), (13)^.

Hearing assessments in mice can be performed quickly by placing two electrodes on the head of the mouse to perform EEG; however, assessing whether detected hearing impairments are congenital requires repeated ABR measurements because deafness cannot be detected via contiguous behavioral observation of the mouse. The ABR measurement system consists of precision apparatus that need careful adjustments to detect peaks with extremely short latencies and avoid increased background noise during ABR measurement, and average 1,000 events to produce accurate waveforms. In this study, we focused on methodological aspects of hearing loss research; specifically, we investigated the effect of background noise on the accuracy of ABR measurement. Using the optimized conditions, we identified several laboratory mouse strain subgroups, including potential congenital hearing impairment models.

## Materials and Methods

### Ethical statement

All experimental protocols were approved by the Committee for Animal Experimentation of the School of Science and Engineering at Waseda University (permission #09A11) and were performed in accordance with the laws of the Japanese government. Adequate measures were taken to minimize pain or discomfort.

### Animals

To measure the effect of background noise on ABR measurement accuracy, 10 randomly selected ICR male mice (Japan SLC, Inc., Shizuoka, Japan) weighing 20-30 g, were used at the age of 8 weeks.

The animals were singly housed in individually ventilated cages (Lab Products, Inc., Seaford, DE, USA), in a room with controlled temperature (22℃ ± 2℃), humidity (60% ± 5%), and light (12/12 h light/dark cycle with lights on at 08:00). The light intensity at the surface of the cages was approximately 100 lux. All mice were on a standard rodent diet (AIN-93M, Research Diets, Inc, USA), and food and water were provided *ad libitum*.

Next, to identify sources of mouse model candidates for congenital hearing loss, we selected 10 male mice from each of the following strains: C57BL/6JJmsSlc (Japan SLC, Inc. Shizuoka, Japan), C57BL/6JJcl (CLEA Japan, Inc., Tokyo, Japan), C57BL/6NCrl (Charles River Laboratories Japan, Inc., Tokyo. Japan), Balb/c (Japan SLC, Inc.), CH3 (CLEA Japan, Inc.), ICR (Japan SLC, Inc.), and ddY (Japan SLC, Inc.).

Mice were tested at 8 weeks of age, and their body weights ranged between 20 and 30 g. Mice were randomly selected from each strain.

All mice remained in the same environmental conditions as described.

In admission, this experiment was approved by the Waseda University laboratory animal ethical review board.

### ABR measurement

**Figure 1.**
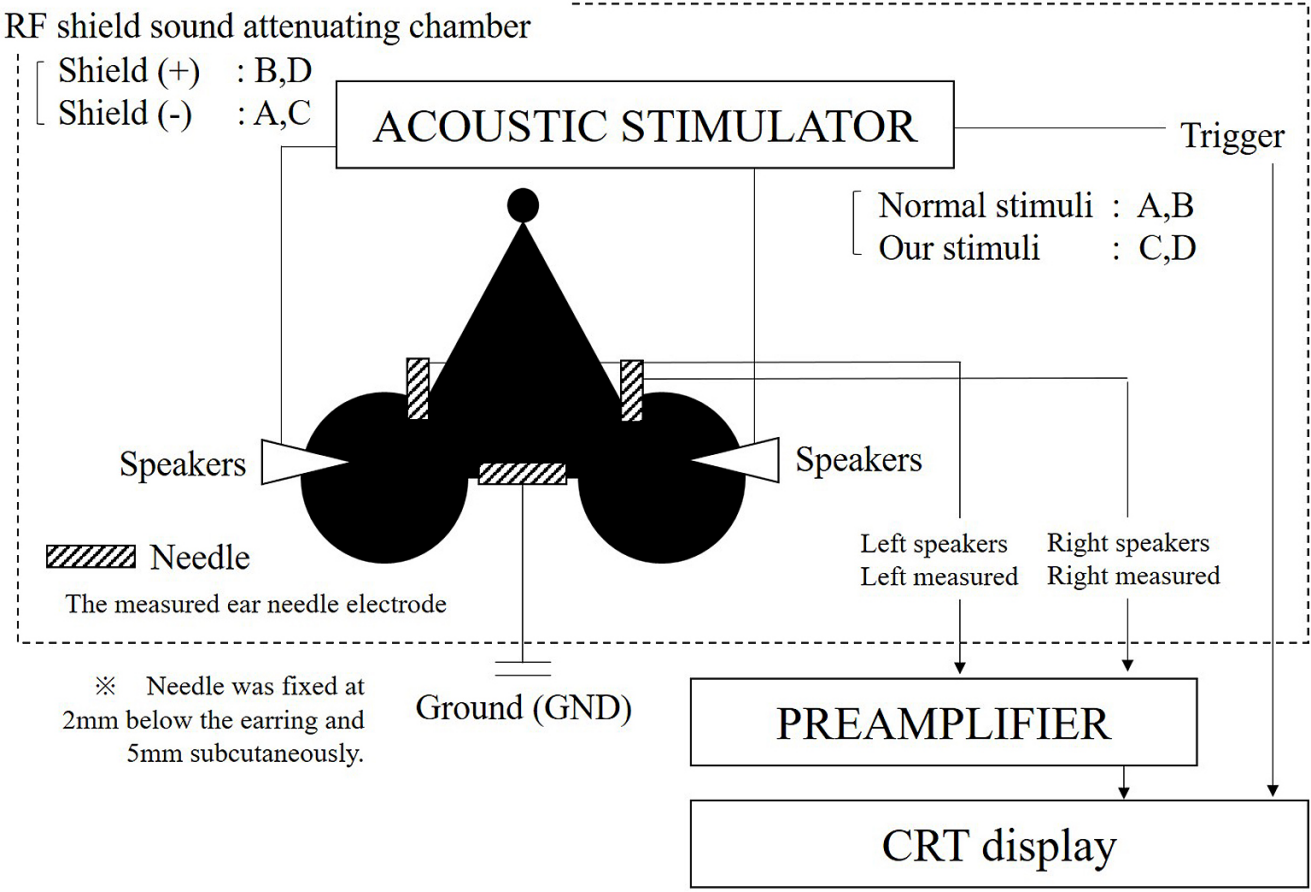
Electrode placement on the mouse’s head for ABR measurement. A, B, C, and D represent the different testing conditions.

ABRs were measured with a 16-kHz tone burst stimulus and recorded with an ABR recording system (Tucker-Davis Technologies: TDT, USA). This system is widely used ^(4)^. Figure 1 shows the placement of the recording system on the head of a test mouse. To fit the recording system, mice were anesthetized with a xylazine hydrochloride solution (0.15 ml/10 grams of body weight) injected intraperitonially. The needle was fixed 2 mm below the earring and 5 mm subcutaneously in the mouse. The measured electrode was set at the earring.

MF1 Multi-Field Magnetic Speakers (Tucker-Davis Technologies: TDT, USA) were attached to the mice, and ABR was measured in closed fields. We also used the tube without attaching it before the MF1 Multi-Field Magnetic Speakers cone.

To calibrate the speakers, the maximal sound pressure of the frequencies used was set to 90 db. The sound pressure measurement amplifier, NA-42, and the capacitor microphone, UC-54 (RION Co. for measurement, Ltd., Tokyo, Japan), was used to measure real sound pressure. An attenuator was used to attenuate the sound pressure.

**Figure 2.**
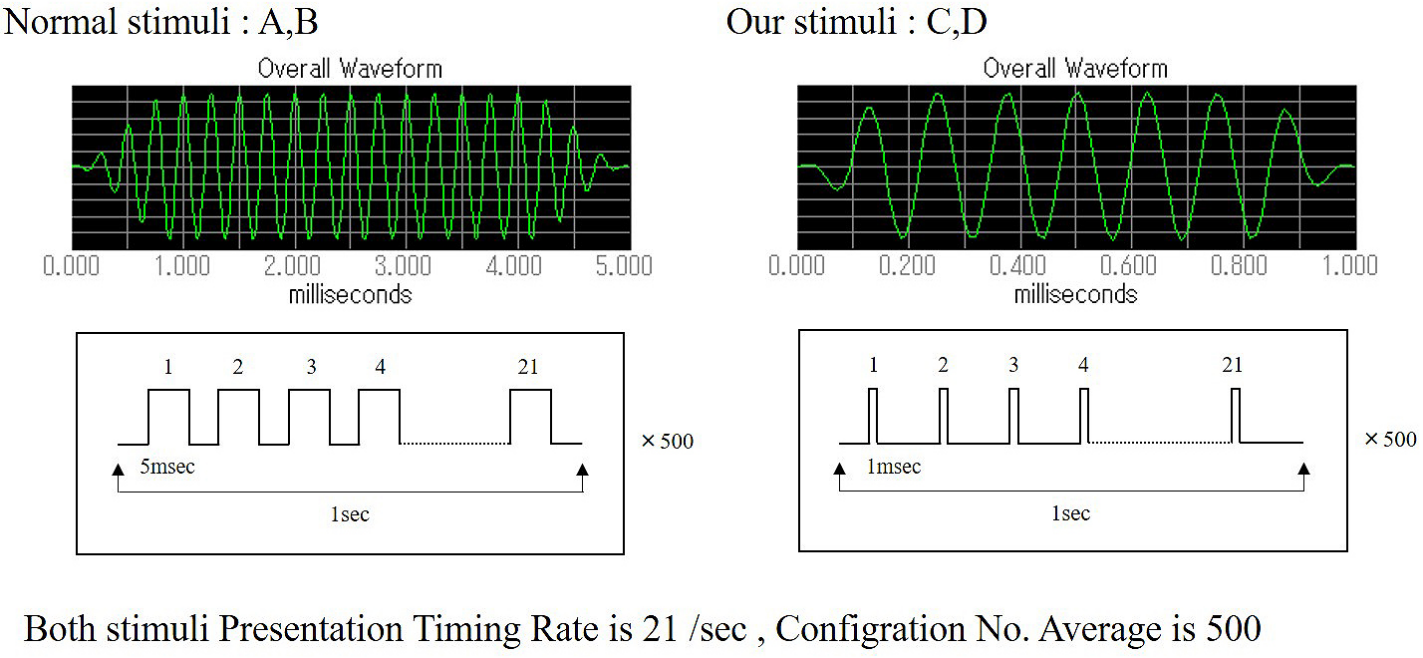
Two types of tone burst waves. Normal stimuli: A, B, consisting of a 5-msec duration with a sound gate of 1 msec. Our stimuli: C, D, consisting of a 1-msec duration with a sound gate of 0.1 msec. Both stimuli presentation timing is 21 times/sec, and configuration number average is 500.

Two types of tone burst were used for this experiment. The conventional tone burst (Nomal stimuli: A, B), consisted of a 5-msec duration with a sound gate（Cos2Gate）of 1 msec. The tone burst duration was 3 msec, and the following sampling time was 5 msec. A newly programmed tone burst (developed by the author, Our stimuli: C, D) consisted of a 1-msec duration with a sound gate (Cos2Gate) of 0.1 msec. Therefore, the tone burst duration was 0.6 msec, and the following sampling time was 9.9 msec. Both stimuli presentation timing was 21 times/sec, and the configuration number average was 500. Our program was developed using the SigGen RP device (Tucker-Davis Technologies: TDT, USA) (Figure 2).

The hearing threshold was calculated by averaging the responses of 1,024 sweeps at each intensity level: 30 dB, 40 dB, 50 dB, 55 dB, 65 dB, 75 dB, and 90 dB (7 steps). The sound to be measured at each level was defined using the BioSig software (Tucker-Davis Technologies). All mice were subjected to measurements in the same way.

### RF shield sound-attenuating chamber

All ABR measurements were performed inside a sound-attenuating chamber (Mitsuihome, Tochigi-Utsunomiya business office, Japan), which was covered by double 0.06-mm aluminum foil and sound-absorbing material. The chamber size was outer 420 mm × 425 mm × 420 mm and inner 240 mm × 300 mm × 240 mm (L × W × H). The chamber’s function as a Faraday cage was confirmed by the interruption of the mobile phone. On the other hand, sound reduction performance is D-40. The Architectural Institute of Japan’s "D value" corresponds to the Dr value of the Japanese Industrial Standard.

### Experimental design

To determine the effect of background noise on ABR measurement accuracy, we used four testing conditions:

A. RF field chamber absent; conventional tone burst

B. RF field chamber present; conventional tone burst

C. RF field chamber absent; new tone burst

D. RF field chamber present; new tone burst

Each mouse was anesthetized and tested once under each test condition. Each trial lasted approximately 10 minutes.

The sound stimulus was presented through a small speaker (EC1/ES1 electrostatic speaker, Patent No. US 6,842,964 B1), which, according to the testing conditions, was placed either inside or outside the chamber. Because waveforms over 75 dB are almost identical, only a 90 dB sound level was used for this experiment.

Next, to identify the strains of mouse model candidates for congenital hearing loss, all mice were tested under testing condition IV from RF field chamber and new tone burst.

Each mouse was tested once for each of the following sound levels: 90 dB, 75 dB, 65 dB, 55 dB, 50 dB, 40 dB, and 30 dB. Each trial lasted approximately 10 minutes, and the sound was presented through a small speaker (EC1/ES1 electrostatic speaker, Patent No. US 6,842,964 B1) placed inside the chamber.

### Competent mice

Following analysis of the hearing threshold of each strain, strains that exhibited a hearing threshold ≤ 30 dB in both ears, with clearly defined wave patterns, were classified as “competent mice,” because congenital hearing loss could be excluded in such mice.

### Wave pattern analysis

**Figure 3.**
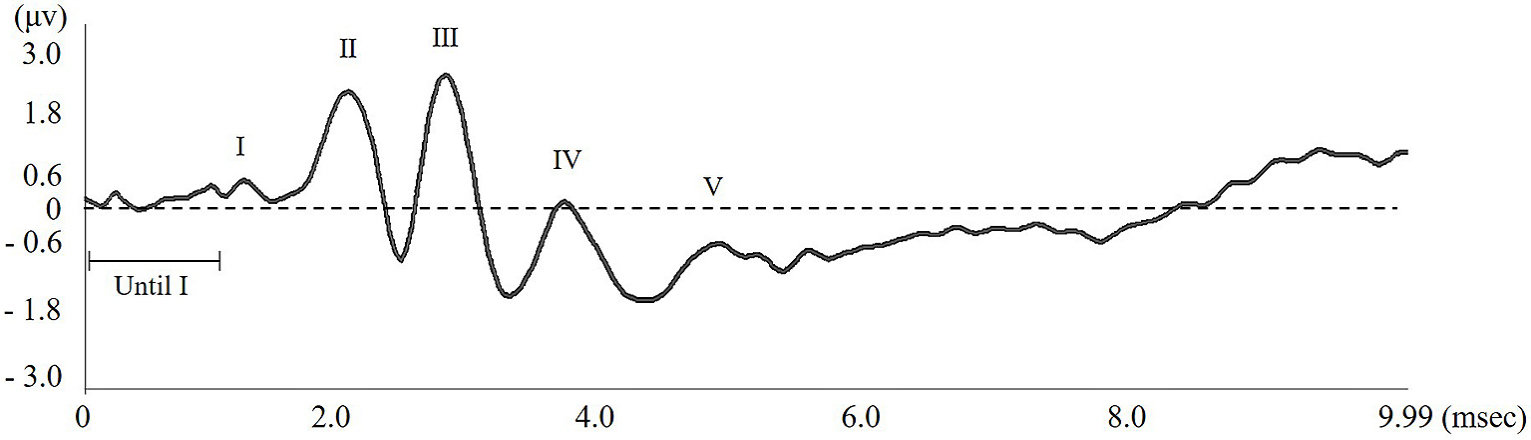
Example of an ABR wave form measured in a ICR mouse. Using a sound-attenuating RF field chamber and a short-tone burst sound resulted in a clearly defined wave. The hearing threshold is calculated from the latency to wave I, the length of the wave pattern and distance between the top and bottom of each wave.

Each wave pattern was analyzed by measuring the length between top and bottom of the wave peak. Lengths of the maximum and minimum latencies, 1 msec before the first wave occurred, were also measured; these were defined as “Until wave I.” Thereafter, any wave pattern occurring when the amplitude was greater than “Until wave I” was recognized as an effective wave pattern (Figure 3).

### Statistical analysis

All data are represented as mean ± SEM (standard error of the mean). GraphPad Prism version 6.03 (GraphPad software, San Diego, CA, USA) was used for statistical analysis. Data were analyzed for normal distribution and homogeneity of variance using the D’Agostino-Pearson/Kolmogorov-Smirnov and F value tests, respectively. One-way analysis of variance (ANOVA), with the Tukey-Kramer test, was used to conduct parametric analysis, and the Kruskal-Wallis/Friedman test, with Dunn’s test for post hoc analysis, was used to perform non-parametric analysis.

## Results

**Figure 4.**
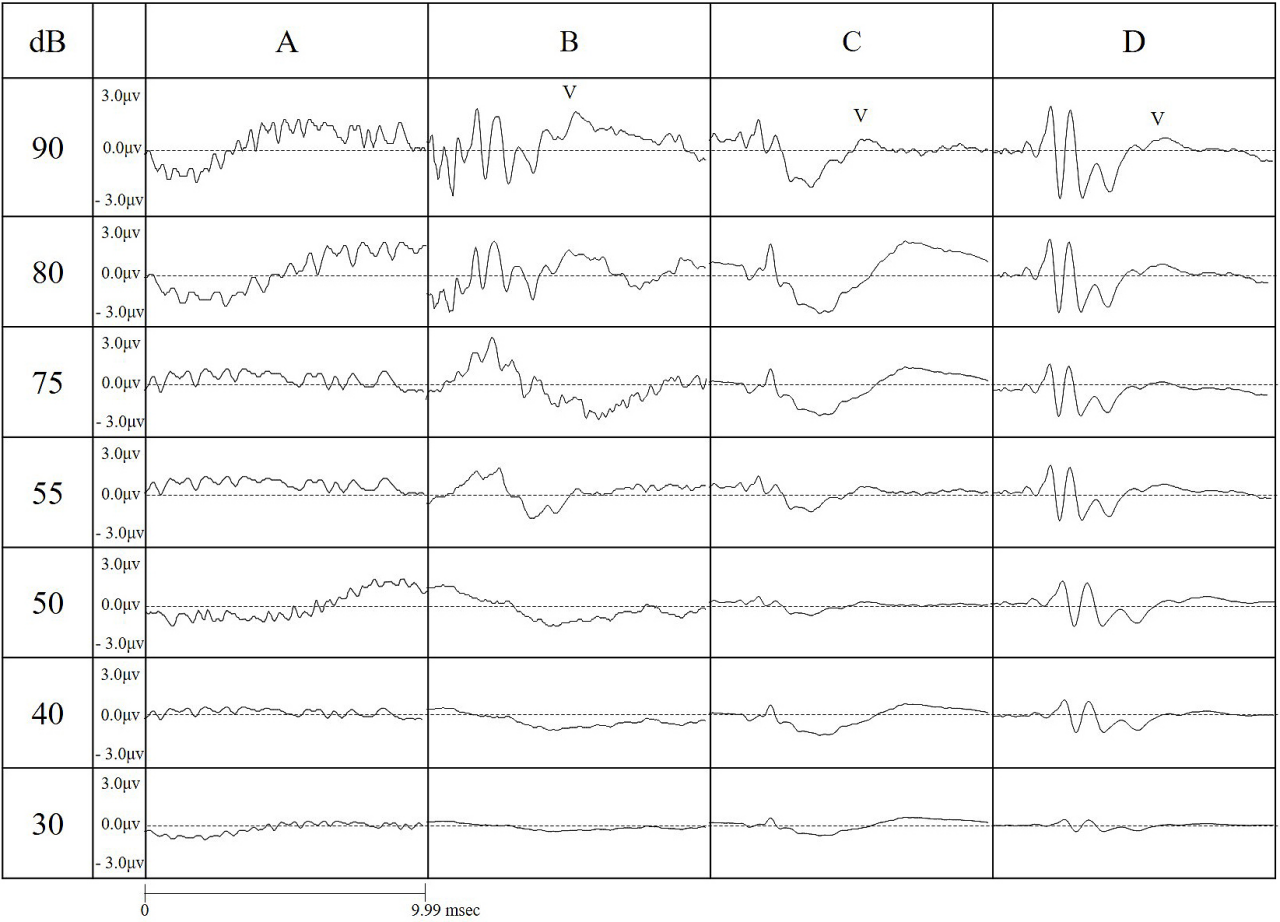
Wave patterns obtained for each sound level at a frequency of 16kHZ under different testing conditions (A - no chamber, conventional tone; B- chamber, conventional tone; C- no chamber, short tone; D - chamber, short tone). The horizontal length corresponds to 4 msec, but the height is −3.0-3.0 μV.

Figure 4 shows the wave length amplitudes, as measured in all conditions. For both short and long tones, using an RF field chamber resulted in a larger wave pattern. In contrast, the shorter tone programmed for this study reduced the noise occurring before wave I compared to the conventional tone. When using the conventional tone, it was difficult to identify wave patterns without using the RF chamber, but the shorter tone allowed a clear identification of the wave pattern both with and without the RF chamber. Using the chamber with the short tone provided the clearest wave pattern. As the strength of the sound stimulus decreased, the time required to identify a wave increased. Without an RF field chamber, wave patterns became indistinct at all decibels, but the RF chamber provided a more distinct wave length, which became optimal with simultaneous use of the short tone.

**Figure 5.**
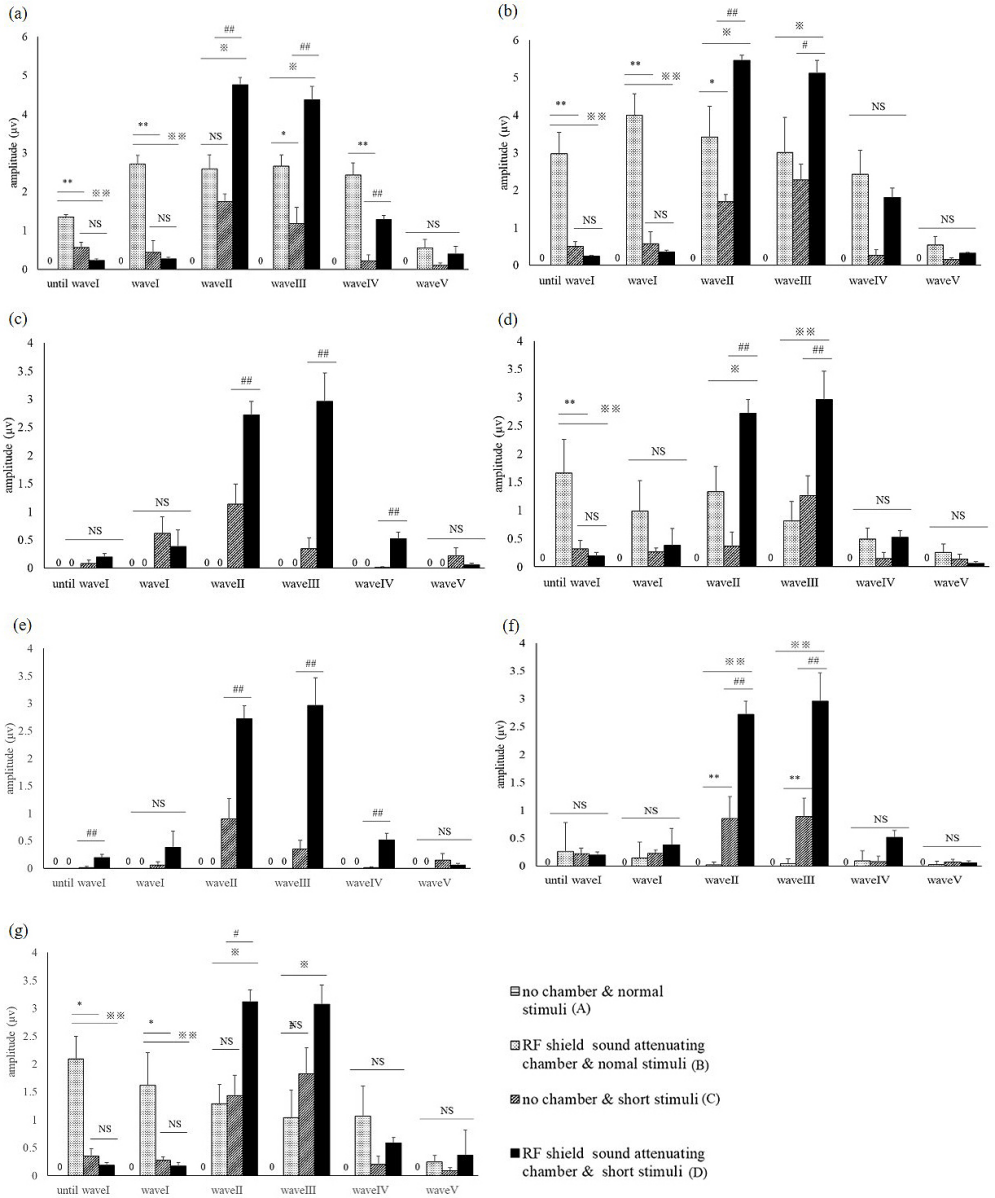
a)-g) Mean amplitude of each wave for the 10 ICR mice, measured at 16kHz frequency and sound levels of (a) 90 dB, (b) 75 dB, (c) 65 dB, (d) 55 dB, (e) 50 dB, (f) 40 dB, and (g) 30 dB. The symbols indicate significant differences in the ANOVA with Tukey-Kramer test. ※※p < 0.01 ※p < 0.05 vs. B, ＊＊p < 0.01 ＊p < 0.05 vs. C, ♯♯p < 0.01 ♯p < 0.05vs. D．

Figure 5 a)-g) shows the average wave pattern for the 10 ICR mice for each sound level, illustrating the absence of an RF field chamber caused a lower wave pattern amplitude, with indistinct wave patterns.

As the sound volume decreased, all wave patterns became attenuated. For a 30-dB sound, identification of all wave patterns was only possible under C and D conditions.

Under testing condition A, no waves were identified; for condition B, there was a clear wave I, but subsequent waves were increasingly difficult to identify; under testing conditions C and D, wave I could be identified, but subsequent waves could only be identified if the RF field chamber was used.

Use of the RF field chamber significantly attenuated the noise until the occurrence of wave I, but did not have a similar effect for subsequent waves (P < 0.01 vs. B or C, Tukey-Kramer test t). For wave I, a high wave pattern amplitude was achieved by using either the RF shield chamber or the short tone (P < 0.01, 0.05 vs. A, Tukey-Kramer test).

For wave II, a higher amplitude was achieved by simultaneous use of the short tone and the RF shield chamber (P < 0.05 vs. A, Tukey-Kramer test). For waves III and IV, a higher amplitude was achieved by simultaneous use of the shorter tone and RF shield chamber (wave III: P < 0.01, 0.05 vs. A-C; wave IV: P < 0.05 vs. A; Tukey-Kramer test), but isolated use of either the chamber or the eshort tone did not provide a similar result (Figure 5 a-g).

The highest accuracy of ABR measurement was achieved under condition D, indicating simultaneous use of both conditions is necessary for an optimal result.

**Figure 6.**
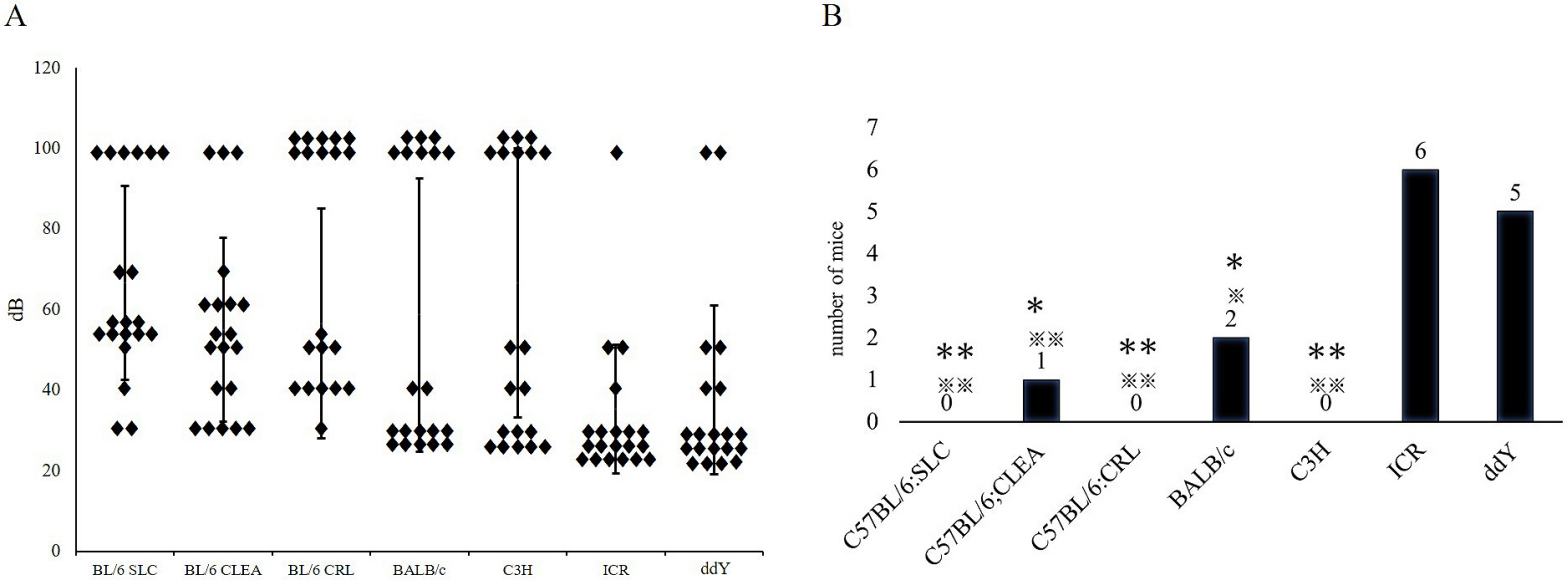
(A) Hearing thresholds measured for each ear in the 10 mice (right ear and left ear), in decibels. (16kHz) The vertical bars show standard deviation. (B) The mean of the number of competent mice (n = 10 8weeks of age, 16 kHz, 90db). Parametric analysis was conducted by Fisher’s exact test, ※※p < 0.01 ※p < 0.05 vs Kwl:ICR ^✽✽^p < 0.01 ^✽^p < 0.05 vs Kwl:ddY.

Identifying strains of mouse model candidates for congenital hearing loss shows the honey bee records present hearing thresholds for 10 mice (right and left ear, n = 20) of each strain (Figure 6A). Table 1 presents hearing thresholds for 10 mice of each strain. In several mice, ABR measurement could not be performed at 90 db.

**Table 1.** Hearing Threshold Measured for Each Ear in Each of the 10 Mice at 16kHz.

	Inbred strains										Closed colony outbred strains			
	C57BL/6						BALB/c		C3H		ICR		ddY	
	SLC		CLEA		CRL		SLC		CLEA		SLC		SLC	
1		55		65		CNM		30		CNM	○	30		30
		55		30		55		CNM		CNM		30		40
2		75		40		CNM		30		30	○	30	○	30
		75		65		40		40		CNM		30		30
3		55		55		40		CNM		30	○	30	○	30
		55		55		40		CNM		CNM		30		30
4		CNM		75		50	○	30		CNM	○	30	○	30
		CNM		50		40		30		30		30		30
5		55		50		50	○	30		30		30		30
		55		30		40		30		40		CNM		CNM
6		30		65		50		40		CNM		30		CNM
		40		30		30		30		30		40		30
7		CNM		65		CNM		CNM		30	○	30	○	30
		CNM		50		CNM		CNM		CNM		30		30
8		50		CNM		CNM		CNM		30	○	30	○	30
		30		40		CNM		30		50		30		30
9		CNM		CNM		CNM		CNM		CNM		50		50
		CNM		CNM		CNM		30		40		30		40
10		55	○	30		CNM		30		30		30		30
		55		30		CNM		CNM		50		50		50

The upper value corresponds to the right ear, whereas the lower value corresponds to the left ear. The acronym CNM indicates instances where a threshold “could not be measured.” The ○ signs indicate subjects classified as “competent mice.”

Within C57BL/6SLC, C57BL/6JCRL, and C3H/CLEA strains, no competent mice were detected; all subjects demonstrated a hearing threshold >30 dB (Figure 6B).

Therefore, these subgroups of strains can be a source of model mice for congenital hearing loss. Within the C57BL/6JCLEA and BALB/cSLC strains, only one and two subjects, respectively, were classified as competent mice, indicating that these subgroups of strains may also serve as congenital deafness models. Conversely, most mice within the ICR/SLC (60%) and ddY/SLC (50%) strains were classified as competent mice, indicating that those subgroups of strains rarely contain models for congenital deafness.

## Discussion

### Use of an RF shield chamber and a shorter tone burst mitigate the effects of background noise

In mouse studies, which have been crucial to understanding the mechanisms of hearing and hearing loss ^(14), (15), (16)^, hearing threshold is typically determined through ABR measurements. However, environmental and methodological factors are likely to affect those measurements’ quality. For example, anesthetizing mice has been shown to decrease ABR measurement accuracy ^(17)^.

Mice hear ultrasonic frequencies, which are not detectable by the human ear ^(18)^. Therefore, when measuring ABR, it is reasonable to hypothesize that background noise, undetectable by humans, may influence ABR measurement accuracy. We investigated the influence of background noise on ABR measurement by comparing the accuracy of measurements performed with and without an RF field sound-attenuating chamber, which allowed us to isolate the animals from environmental electromagnetic waves.

ABRs are typically measured via presentation of a tone burst with 5-msec duration and 1-msec sound gate. A previous study found that increasing the stimulus rate, through presentation of shorter tone bursts, could improve threshold assessment ^(19)^. In our study, we programmed a new, shorter tone burst with a 1-msec duration and 0.1-msec sound gate, to investigate how this might affect ABR measurements’ accuracy.

Our results show that using an RF field chamber increases measurement accuracy for wave I; however, this enhancement effect decreases for subsequent waves. Notably, the simultaneous use of the RF chamber and the new, shorter tone burst, allowed clear separation of waves I-V, even for lower sound intensities.

Van Looij et al. reported ^(17)^ that measuring ABR in anesthetized mice reduced the accuracies of peak and interpeak latencies as well as thresholds; they recommended using awake mice. However, using awake mice involves significantly longer recording times due to myogenic noise. Improving the measurement methodology by reducing background noise and presenting shorter tone bursts may avoid the detrimental effects of anesthesia, making accuracy comparable to that obtained in awake mice.

Typically, clear wave patterns can be obtained with an 80-dB sound, whereas those obtained at 10-20 dB are largely indistinct. When the resulting ABR wave patterns are unclear, the measurements must be repeated several times, which is time-consuming, burdensome to the subject, and yields a lower amplitude of detected wave patterns. Simultaneous use of a sound-attenuating chamber and a shorter tone avoids these issues.

ABR waveform does not oscillate significantly with the different anesthetic regimens used.

For example, since our basic experiments have shown that inhalation anesthesia, such as isoflurane, causes less ABR and delays in waveform than celiac anesthesia, such as pentobarbital, we use anesthesia that has less effect on waveform and the mouse’s body.

Further studies are needed to investigate how this methodology can be transposed into the clinical setting for applying ABR measurements of human patients.

### Mouse models of congenital hearing loss

More than 400 hearing-impaired mutant mouse models have been identified amongst laboratory mice populations ^(8)^. While several suitable models are available to study age-related hearing loss ^(7)^, identifying good models for congenital hearing loss requires different testing procedures since the onset of hearing loss is early in a mouse’s life.

We investigated the hearing threshold and ABR pattern of several commonly used mouse strains during early adulthood (eight weeks). Within each strain, mice with normal or close to normal hearing were classified as “competent mice,” while those not meeting this criterion were regarded as exhibiting potential congenital hearing loss. A greater proportion of “competent mice” was found within the closed colony outbred strains, indicating that potential congenital hearing loss models are more easily found among inbred strains. Interestingly, congenital deafness in many species is often associated with the loci of white pigmentation ^(20)^. Conversely, in laboratory mice, it mostly occurred among C57BL/6 sub-strains, whereas 20% of the BALB/C mice revealed a normal hearing threshold; the outbred strains were both albino. Notably, some strains (e.g., ICR/SLC) are less likely to exhibit congenital hearing loss and are more suitable for studying hearing function.

Although unrelated to coat pigmentation, our results regarding hearing in young adulthood are consistent with previous, ground-breaking research performed in older mice, which showed that several inbred strains exhibit hearing impairments from middle age, unlike F1 hybrids ^(14), (21), (22)^. Those previous studies concluded that inbred strains are good models of age-related hearing research and identified the genes contributing to this condition ^(23)^; however, our results suggest that some strains may be suitable congenital hearing loss models, although further studies are needed to consolidate this view and investigate underlying mechanisms.

This study does not allow reference to the functional analysis of mouse genetic background and deafness-related genes. However, it is of great help in selecting mice for ABR measurements.

### Conclusion

Environmental factors, such as background noise, exert an influence on ABR measurements’ accuracy. Improving measurement methodology by addressing these kinds of factors will lead to enhanced use of animal models of hearing loss, with consequent understanding of human hearing pathologies.

A several inbred, commercially available mouse strains are potential models for congenital hearing loss; further research is needed to clarify mechanisms involved in early-onset hearing loss.

## Article Information

### Conflicts of Interest

None

### Sources of Funding

This work was supported by the Asama Institute (grant number AIGNO0001).

### Acknowledgement

The authors thank Mr. Shinji Kume from the Bio Research Center Co., LTD. for his valuable comments.
